# Tin(II) Dithiocarbamate-Derived SnS Nanoparticles for High-Performance Quantum Dot-Sensitized Solar Cells

**DOI:** 10.3390/nano16120718

**Published:** 2026-06-10

**Authors:** Inam Vulindlela, Athandwe M. Paca, Edson L. Meyer, Mojeed A. Agoro, Nicholas Rono

**Affiliations:** 1 Fort Hare Institute of Technology, University of Fort Hare, Private Bag X1314, Alice 5700, South Africa; 201614310@ufh.ac.za (I.V.); emeyer@ufh.ac.za (E.L.M.);; 2 Department of Chemical and Earth Science, Chemistry Discipline, University of Fort Hare, Private Bag X1314, Alice 5700, South Africa; apaca@ufh.ac.za; 3 Department of Basic Sciences, Tharaka University, Marimanti P.O. Box 193-60215, Kenya

**Keywords:** SnS quantum dots, dithiocarbamate precursors, quantum dot-sensitized solar cells, monofacial and bifacial, power conversion efficiency

## Abstract

The increasing global demand for renewable energy has intensified the search for high-efficiency and cost-effective solar cell technologies. Quantum dot-sensitized solar cells (QDSSCs) have emerged as promising candidates due to their tunable optoelectronic properties and enhanced light absorption. In this study, SnS quantum dots were synthesized from dithiocarbamate complexes using different ligands, namely *m*-toluidine (SnS1), aniline (SnS2), and *p*-toluidine (SnS3), to investigate the influence of precursor chemistry on material properties and device performance. Structural analysis confirmed the formation of an orthorhombic phase for all samples, while morphological studies revealed well-dispersed nanocrystals for SnS1 (5.93 nm), increased aggregation for SnS2 (8.57 nm), and partially fused domains with an intermediate size for SnS3 (6.67 nm). Optical measurements showed bandgap energies of 2.8, 2.2, and 2.7 eV for SnS1, SnS2, and SnS3, respectively, with SnS3 exhibiting reduced charge-recombination behaviour. Photovoltaic devices fabricated using these materials yielded power conversion efficiencies of 3.40, 2.03, and 7.63% for SnS1, SnS2, and SnS3, respectively, with no significant improvement observed for bifacial configurations. The superior performance of SnS3 is attributed to an optimal balance between light absorption, morphology, and charge transport properties, highlighting the critical role of precursor ligand selection in tuning quantum dot characteristics for improved QDSSC performance.

## 1. Introduction

Recent years have seen a significant increase in interest in metal sulfide semiconductor nanostructures because of their remarkable physicochemical characteristics. Among the IV–VI group semiconductors, germanium sulfide (GeS), tin sulfide (SnS), and lead Sulfide (PbS) have attracted considerable interest because of their suitability for use in photo transducers, infrared sensors, photovoltaic systems, and other optical applications [1,2,3]. Tin sulfide in particular has received growing attention as a promising, low-cost photovoltaic material, owing to its desirable features such as a direct optical band gap of about 1.3 eV, a high absorption coefficient in the visible region (>10^4^ cm^−1^), good carrier mobility (~30 cm^2^V^−1^s^−1^), and intrinsic p-type electrical conductivity [2,4]. Furthermore, tin and sulfur are both affordable, less hazardous, and abundant components of the Earth. Tin sulfide nanoparticles can exhibit either p-type or n-type conduction, depending on the tin concentration [1,4]. In addition, the flexible coordination behaviour of tin and sulfur allows tin sulfide to form multiple phases, including SnS, Sn_2_S_3_, Sn_3_S_4_, and SnS_2_, contributing to its diverse structural and electronic properties [4,5,6].

Various methods have been reported for the synthesis of SnS nanoparticles, including solvothermal, hydrothermal, ionothermal, microwave-assisted, aqueous solution techniques, and single-source precursor approaches [4,7,8,9,10,11,12]. Among these, the single-source precursor method is particularly promising because it allows precise control over the nanoparticle composition and morphology, often produces high-purity materials, and can be more efficient and reproducible compared to multi-precursor techniques [13,14,15]. Rajasekhar et al. [4] demonstrated that the single-source precursor method can produce high-purity, nearly spherical SnS nanoparticles with an orthorhombic crystal structure, where the growth is likely influenced by an oriented attachment mechanism. Furthermore, Ahmet et al. [16] successfully deposited phase-pure SnS thin films using a single-source precursor, (dimethylamido)(N-phenyl-N′,N′-dimethylthiouriate) tin(II) dimer, via aerosol-assisted CVD (AA-CVD). This method avoided the need for volatile precursors and allowed phase control—orthorhombic (α-SnS) at 375 °C and zinc blende (ZB-SnS) at 300 °C. The dense, polycrystalline films showed direct band gaps of 1.34 eV (α-SnS) and 1.78 eV (ZB-SnS), with the latter exhibiting ambipolar behaviour for the first time. Overall, single-source precursor routes have proved to be an efficient and reliable approach for producing high-quality, stoichiometric SnS nanoparticles with controlled morphology and phase purity.

In this work, [Sn(L1)_2_], [Sn(L2)_2_], and [Sn(L3)_2_] complexes were used as single-source precursors to synthesize SnS quantum dots (SnS1, SnS2, and SnS3) using hexadecylamine as a capping agent. The study focuses on how variations in ligand structure influence the formation, optoelectronic properties, and photovoltaic performance of the resulting quantum dots. By tuning the precursor chemistry, this work demonstrates a simple and effective way to control the properties of SnS absorbers for quantum dot-sensitized solar cell applications. In addition, both monofacial- and bifacial-device configurations are explored, to assess the effect of illumination conditions on device performance. Overall, this study provides useful insight into how precursor design can be used to improve the efficiency of SnS-based QDSSCs.

## 2. Methodology

### 2.1. Materials, Methods and Physical Measurements

All chemicals and reagents were used at analytical grade, without any purification. The primary amines that were used in this study were aniline and m-toluidine. Additional chemicals that were utilized included ammonia, carbon disulfide, diethyl ether, methanol, tin(II) chloride, dimethyl sulfoxide (DMSO), dexadecylamine (HDA), oleic acid (OA), and chloroform. Complete testing kits, comprising FTO/TiO_2_ substrates, Pt-FTO counter electrodes, HI-30 iodide electrolyte, masks, gaskets, chenodeoxycholic acid (CDC), and hot seals, were obtained from Solaronix (Aubonne, Switzerland). The ligands were synthesized according to a modified literature procedure [17]. *N*-phenyldithiocarbamate (L_1_) Yield: 32%, Colour: yellow-orangish, Molecular weight (g/mol): 186.31, Molecular formula: C_7_H_10_N_2_S_2_, M.pt: 130–140 °C, FTIR/KBr, cm^−1^: 3418 (C-H), 1490 (C-N), 1072 (C=S). UV–Vis (CH_3_OH solution, nm): 250–340, 375–382. *N*-(3-methylphenyl)dithiocarbamate (L_2_) Yield: 68%, Colour: yellowish white, Molecular weight (g/mol): 201.3, Molecular formula: C_8_H_13_N_2_S_2_, M.pt: 130–140 °C, FTIR/KBr, cm^−1^: 3370 (C-H), 1508 (C-N), 1083 (C=S). UV–Vis (CH_3_OH solution, nm): 265–300, 350–390. *N*-(4-methylphenyl)dithiocarbamate (L_3_) Yield: 68%, Colour: yellowish white, Molecular weight (g/mol): 201.3, Molecular formula: C_8_H_13_N_2_S_2_, M.pt: 130–140 °C, FTIR/KBr, cm^−1^: 3370 (C-H), 1510 (C-N), 1082 (C=S). UV–Vis (CH_3_OH solution, nm): 250–340, 375–380.

### 2.2. Synthesis of Sn(II) Dithiocarbamate Complexes

A solution of SnCl_2_ (0.28 g) in distilled water was added separately to aqueous solutions of aniline dithiocarbamate (0.932 g), m-toluidine dithiocarbamate (1.00 g) and p-toluidine dithiocarbamate (1.00 g) in a 2:1 ligand-to-metal ratio, with continuous stirring, at room temperature, for 2 h, leading to the formation of mustard, light-yellow, and yellow precipitates, respectively. All precipitates were filtered, washed thoroughly with distilled water, and dried in a fume hood. The resulting complexes were formulated as [Sn(L_1_)_2_] Yield: 78%, Colour: mustard, Molecular weight (g/mol): 455.25, Molecular formula: C_14_H_12_N_2_S_4_Sn, M.pt: 150–240 °C, FTIR/KBr, cm^−1^: 3171 (C-H), 1504 (C-N), 671 (C-S), 467 (Sn-S). UV–Vis (CH_3_OH solution, nm): 260–340. Raman, cm^−1^: 1007 (C=S), 676 (C-S), 423 (Sn-S)., [Sn(L_2_)_2_] Yield: 78%, Colour: light yellowish, Molecular weight: 485.31, Molecular formula: C_16_H_18_N_2_S_4_Sn, M.pt: 150–250 °C, FTIR/KBr, cm^−1^: 3183 (C-H), 1492 (C-N), 980 (C=S), 486 (Sn-S). UV–Vis (CH_3_OH solution, nm): 260–330. Raman, cm^−1^: 1000 (C=S), 670 (C-S), 423 (Sn-S)., and [Sn(L_3_)_2_] Yield: 78%, Colour: yellow, Molecular weight: 485.31, Molecular formula: C_16_H_18_N_2_S_4_Sn, M.pt: 150–250 °C, FTIR/KBr, cm^−1^: 3185 (C-H), 1492 (C-N), 982 (C=S), 486 (Sn-S). UV–Vis (CH_3_OH solution, nm): 260–330. Raman, cm^−1^: 1000 (C=S), 672 (C-S), 423 (Sn-S).

### 2.3. Synthesis of SnS Quantum Dots

In a typical synthesis, 3 g of hexadecylamine was introduced into a three-necked flask fitted with a thermometer, reflux condenser, and rubber septum and heated under a nitrogen atmosphere to 200 °C with continuous stirring. Separately, each precursor complex, [Sn(L_1_)_2_], [Sn(L_2_)_2_], and [Sn(L_3_)_2_] (0.25 g), was dispersed in 6 mL of oleic acid and swiftly injected into the hot Hexadecylamine solution using a syringe, based on the literature [18]. Following injection, the temperature dropped by approximately 15–30 °C, but was maintained at 200 °C for 1 h to ensure complete reaction. After cooling the mixture to about 70 °C, cold ethanol was added to quench the reaction, and the nanoparticles were collected by centrifugation at 2000 rpm for 30 min. Repeated washing with cold ethanol removed residual surfactants, and the products were dried under vacuum. The resulting quantum dots, prepared from [Sn(L_1_)_2_], [Sn(L_2_)_2_], and [Sn(L_3_)_2_], were labelled SnS1, SnS2, and SnS3, respectively.

### 2.4. Solar-Cell Fabrication and Assembly

Quantum dot-sensitized solar cells (QDSSCs) were fabricated using 2 × 2 cm^2^ FTO glass substrates with TiO_2_ electrodes (6 × 6 mm^2^ active area) obtained from Solaronix (Aubonne, Switzerland) and platinum-coated FTO as counter electrodes. For SnS1, 0.015 g of the synthesized quantum dots was dissolved in 10 mL of chloroform, and the TiO_2_ electrode was immersed in this solution for 24 h to allow proper sensitization. After quantum dot loading, the photoanode and counter electrode were placed directly together in a sandwich arrangement, with their active sides facing each other, and the device was held firmly in place using clips on both sides. The assembled cell was then injected with the commercial HI-30 iodide electrolyte (0.05 M, Solaronix, Aubonne, Switzerland), to complete the device. The same procedure was repeated for SnS2 and SnS3.

### 2.5. Characterization

UV-Vis spectroscopy (PerkinElmer Lambda 365, Gauteng, South Africa) was used to record all the electronic spectra of complexes and ligands dissolved in a solvent (DMSO) recorded over a wavelength range of 200–800 nm PerkinElmer LS 45 fluorescence (Gauteng, South Africa) was used to record PL spectra of nanoparticles that were dissolved in chloroform. The excitation wavelength was set at 400 nm. PerkinElmer-2000, Gauteng, South Africa was used to record all FTIR spectra with a KBr pellet technique. Thermal decomposition of Tin Sulfide nanoparticles was performed on Universal V4.5A TA instruments, Gauteng, South Africa over a temperature range of 30–900 °C, at a heating rate of 10 °C/min, under a nitrogen (N_2_) atmosphere. XRD measurements were conducted using a Bruker D8 Advance diffractometer with Cu Kα radiation (λ = 1.5406 Å) over a 2θ range of 10–80° with a step size of 0.02°, to determine the crystallite size and phase of the nanoparticles. Atomic force microscopy (AFM, JPK NanoWizard II, JPK Instruments, Berlin, Germany) in contact mode at a scan rate of 0.8 Hz was employed to evaluate the surface roughness of the SnS-modified FTO substrates. Transmission electron microscopy (TEM) images were recorded using a JEOL JEM-1400 electron microscope to examine the morphology and size of the nanoparticles. The Zeiss EVO LS 15 scanning electron microscope, equipped with EDS INCA Penta FET x3 at 20.00 kV with 23 mm WD, was used to obtain the SEM images and EDS spectra. A confocal Raman AFM imaging system (WITec GmbH) alpha300RS was used to acquire the materials’ Raman spectra. Electrochemical characterization was carried out using cyclic voltammetry (CV) at scan rates ranging from 0.05 to 0.35 V s^−1^ in 0.05 V s^−1^ increments. Electrochemical impedance spectroscopy (EIS) measurements were performed over a frequency range of 100 kHz to 100 mHz. Current–voltage (J–V) characteristics were recorded using a Keithley 2401 source meter in conjunction with a Thorax light power meter (RS Components, Johannesburg, South Africa). A Lumixo AM1.5 solar simulator provided illumination, with the lamp positioned 50 cm above the sample to ensure uniform exposure of the working area. To prevent cell degradation, the temperature was maintained below 60 °C, and the incident light power density was set at 100 mW cm^−2^ (AM1.5).

## 3. Results and Discussion

### 3.1. FTIR and Electronic Spectra Studies of the Ligands

The FTIR spectra of the ligands synthesized in this work show three primary characteristic bands are observed in all dithiocarbamate ligands, corresponding to N–H, C–N, and C=S/C–S vibrations, which are critical for coordinating metal ions in the complexes [19]. Specifically, Ligand 1 exhibits bands at 3418, 1490, 1072, and 996 cm^−1^, Ligand 2 shows bands at 3371, 1508, 1083, and 999 cm^−1^, and Ligand 3 shows bands at 3370, 1510, 1082, and 999 cm^−1^, assigned to *ν*(N–H), *ν*(C–N), *ν*(C=S), and *ν*(C–S), respectively, corresponding to the same functional group vibrations. The presence of these features confirms the successful formation of the dithiocarbamate ligands with nitrogen and sulfur donor atoms suitable for coordination. Minor variations in peak positions among the ligands are attributed to differences in substituent effects and local electronic environments. These assignments are consistent with previously reported studies [19,20]. Furthermore, UV–Vis spectra of the synthesized ligands show two prominent absorption regions. The first, a higher-energy band, observed at 265–300 nm for L1, and 250–340 nm for L2 and L3, is attributed to π→π* transitions within the conjugated ligand framework. The second, a lower-energy band, appearing at 350–390 nm for L1, and 375–382 nm for L2 and for L3, corresponds to n→π* transitions involving the lone-pair electrons of nitrogen and sulfur atoms. The observed shifts in these absorption bands reflect differences in the electronic environment of the ligands, influenced by their substituents and molecular structure. These spectral features are in good agreement with literature reports by Zaldi et al. [21], further supporting the successful synthesis of the ligands.

### 3.2. FTIR, Raman, and UV–Vis Analyses of the Sn(II) Complexes

The FTIR spectra of the Sn(II) complexes showed characteristic bands, confirming coordination through nitrogen and sulfur atoms. [Sn(L1)_2_] revealed peaks at 3171, 1504, 671, and 467 cm^−1^, [Sn(L2)_2_] at 3183, 1492, 980, 678, and 486 cm^−1^, and [Sn(L3)_2_] at 3185, 1492, 982, 486, and 474 cm^−1^. The broad band near 3171–3185 cm^−1^ corresponds to ν(N–H), while those around 1490–1504, 974–982, and 671–678 cm^−1^ are attributed to *ν*(C–N), *ν*(C=S), and *ν*(C–S), respectively. The low-frequency absorptions at 467–486 cm^−1^ correspond to ν(Sn–S) vibrations, confirming complex formation and bidentate coordination of the dithiocarbamate ligands, and Agoro et al. [17] also reported similar observations. Raman spectra further supported the FTIR results. [Sn(L1)_2_], [Sn(L2)_2_], and [Sn(L3)_2_] showed bands around 1000–1013, 670–682, and 423–425 cm^−1^, corresponding to C=S, C–S, and Sn–S stretching modes, respectively [22]. The Sn–S vibrational bands observed in all the complexes show that tin(II) is coordinated through the sulfur atoms, confirming the formation of the tin(II) dithiocarbamate complexes. Furthermore, the optical properties of the Sn(II) complexes were further examined using UV–Vis spectroscopy. The UV–Vis spectra showed absorption bands at 260–340 nm for [Sn(L1)_2_], and 260–330 nm for [Sn(L2)_2_] and [Sn(L3)_2_], which are attributed to π→π* and n→π* transitions within the ligand background [21]. The observed emission behaviour reflects ligand-to-metal charge transfer (LMCT) processes and variations in the electronic environments of the complexes, confirming successful complexation and distinct photophysical characteristics [23].

### 3.3. Thermal Stability and Decomposition of Sn(II) Complexes

**Figure 1:**
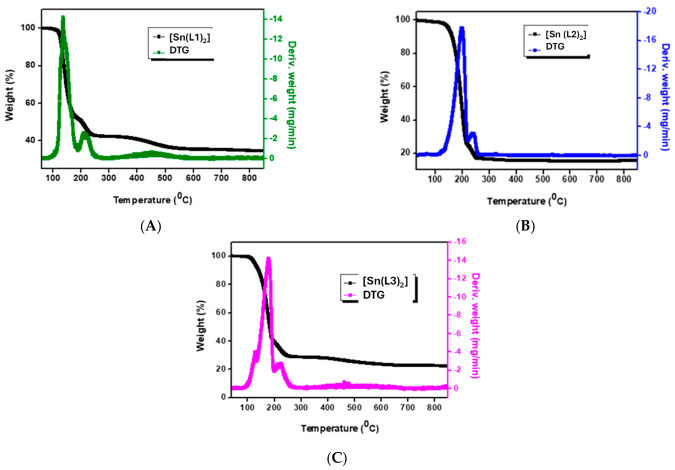
TGA curves of the Sn(II) complexes: [Sn(L1)_2_] (**A**), [Sn(L2)_2_] (**B**), and [Sn(L3)_2_] (**C**).

The thermal stability of the Sn(II) complexes was investigated by TGA and DTG (Figure 1A–C). The [Sn(L1)_2_] showed an initial weight loss from 150 to 238 °C, decreasing to 52%, followed by a gradual decomposition up to 490 °C, leaving 37.38% residual mass. DTG analysis revealed a major peak at 141 mg/min and a minor peak at ~215 mg/min, consistent with stepwise ligand dissociation and partial degradation of the SnS core. In contrast, the [Sn(L2)_2_] complex exhibited a single-step decomposition between 150 and 248 °C, with a residual mass of 16% up to 850 °C. The DTG curve showed a major peak at 204 mg/min and a minor peak at 242 mg/min, suggesting a relatively straightforward loss of coordinated L2 ligands. The [Sn(L3)_2_] displayed a sharp weight loss from 150 to 246 °C to 30% of the original mass, remaining stable up to 850 °C, with DTG peaks at 180 mg/min (major) and 227.7 mg/min (minor). The enhanced thermal stability of [Sn(L3)_2_], relative to the mononuclear complexes, can be attributed to the stabilizing effect of ligand coordination, which likely reinforces the Sn–S coordination framework and hinders decomposition. These results highlight the significant role of ligand environment in modulating the thermal behaviour of SnS complexes.

The thermal stability of the Sn(II) complexes, as revealed by TGA, confirms their suitability for quantum dot formation upon decomposition. The observed weight losses correspond to the removal of coordinated ligands and subsequent formation of SnS as the stable residue. Building on these results, the SnS nanoparticles obtained from these complexes were further characterized to investigate their optical, structural, morphological, and electrochemical properties.

### 3.4. Structural Characterization of SnS Quantum Dot

#### 3.4.1. XRD, Raman and AFM Analysis

**Figure 2:**
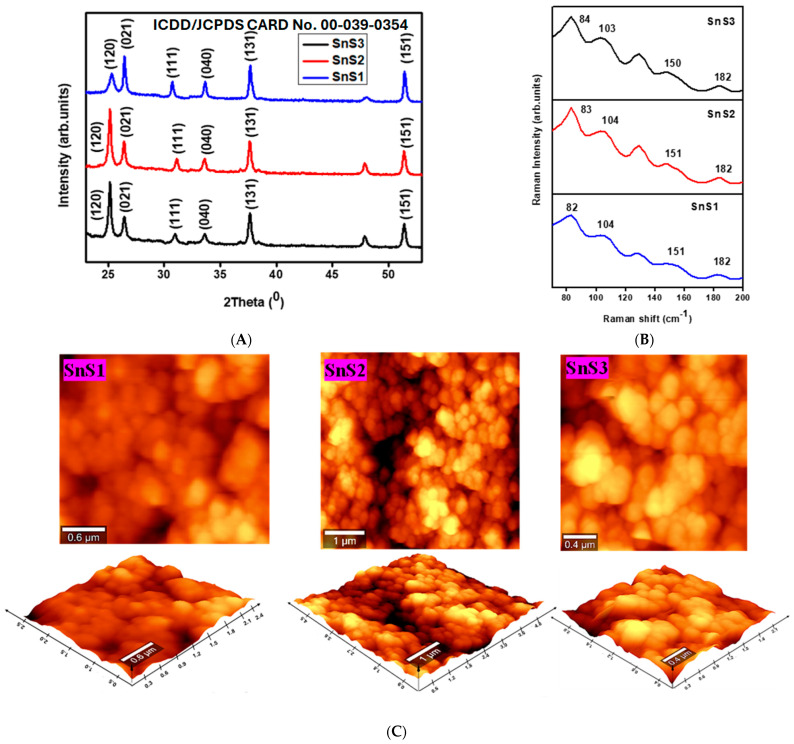
XRD patterns (**A**) of the three SnS samples, their corresponding Raman spectra (**B**), and AFM surface morphology images (**C**).

The XRD patterns of the SnS samples (SnS1, SnS2, and SnS3) exhibit the characteristic orthorhombic SnS phase as shown in Figure 2A, consistent with the work reported by [24], with all samples showing well-defined diffraction peaks indexed to the (120), (021), (111), (040), (131), and (151) crystallographic planes according to the standard ICDD/JCPDS card No. 00-039-0354. The diffraction peaks located at approximately 2θ ≈ 26°, 31°, 38°, 43°, 51°, and 54° correspond respectively to the (120), (021), (111), (040), (131), and (151) reflections of orthorhombic SnS [24]. The good agreement between the experimental peak positions and the standard data confirms successful formation of phase-pure SnS [24,25]. The corresponding XRD parameters, including peak positions (2θ), interplanar spacings (d), full width at half maximum (FWHM), and crystallite sizes for SnS1–SnS3, are summarized in Table 1, further confirming the phase purity and crystalline nature of the prepared samples. The diffraction peaks of the SnS samples exhibit noticeable broadening, which can be attributed to two main factors: nanoscale crystallite size and lattice strain. According to the Scherrer equation, smaller crystallites lead to broader diffraction peaks because the coherent scattering domains are limited in size. Notably, SnS2 and SnS3 display their most intense peak at the (120) reflection, indicating a preferred orientation along this plane, which has been similarly reported in the literature for SnS nanostructures synthesized under conditions promoting anisotropic growth [26]. In contrast, SnS1 exhibits a dominant peak at the (021) plane, suggesting that the ligand environment used during synthesis influenced the nucleation and growth pathway, thereby modifying the preferential crystallographic orientation, a trend also observed by previous studies where ligand type or concentration altered surface energies and growth kinetics of SnS nanoparticles [27]. The overall sharp and intense peaks across all samples reflect high crystallinity, while the slight variations in peak intensity distribution among the samples further confirm structural modulation driven by different ligand interactions [28,29]. These findings align with earlier reports demonstrating that SnS retains its orthorhombic phase regardless of precursor pathway, but the relative growth direction and crystallite orientation can shift, depending on the coordinating ligands involved during synthesis. Additionally, Raman spectroscopy was employed to further confirm the structural features of the SnS nanoparticles and to assess phonon behaviour across the three samples. All samples (SnS1–SnS3) exhibit the characteristic low-frequency vibrational modes of orthorhombic SnS, consistent with the 12 Raman-active phonons predicted for this phase, as Figure 2B illustrates. SnS1 shows distinct peaks at 82, 104, 151 and 182 cm^−1^, while SnS2 displays similar features at 83, 104, 151 and 182 cm^−1^. SnS3 presents slightly shifted modes at 84, 103, 150 and 182 cm^−1^. These bands correspond to the expected B_1_g/B_2_g and Ag/B_3_g lattice vibrations typically observed in nanostructured SnS. The close correspondence of these peaks with those reported by Chowdhury et al. [30], who observed similar Raman modes for SnS nanoparticles, confirms the successful formation of orthorhombic SnS in our samples. The slight shifts in the vibrational modes are attributed to ligand-dependent variations in particle size, lattice strain, and local bonding environments induced by the different precursors, while the overall preservation of peak positions confirms that the crystal structure remains unchanged [31,32]. Furthermore, AFM analysis shows distinct variations in the surface morphology of the SnS thin films (Figure 2C). SnS1 exhibits the smoothest and most compact surface (Ra = 0.35 µm, Rq = 0.47 ± 0.04 µm, Rz = 0.15 µm), indicating uniform grain packing. SnS2 displays the highest roughness (Ra = 0.63 µm, Rq = 0.66 ± 0.03 µm, Rz = 0.22 µm), consistent with larger grains and more pronounced height variations. SnS3 shows intermediate roughness values (Ra = 0.50 µm, Rq = 0.57 ± 0.02 µm, Rz = 0.18 µm), reflecting a moderately textured surface. The smoother and more compact surface of SnS1 may facilitate improved interfacial contact and efficient charge-carrier transport by minimizing surface defects and trap-assisted recombination [33]. In contrast, the higher roughness observed for SnS2 could increase surface irregularities and defect-assisted recombination pathways, thereby hindering efficient charge extraction. SnS3, with intermediate roughness, may provide a balance between surface texturing and interface uniformity. These morphological variations are likely to contribute to the differences observed in the optoelectronic and photovoltaic performance discussed in the subsequent sections, supporting the structural differences observed from XRD and Raman analyses.

#### 3.4.2. TEM, Size Distribution, SEM and EDS

**Figure 3:**
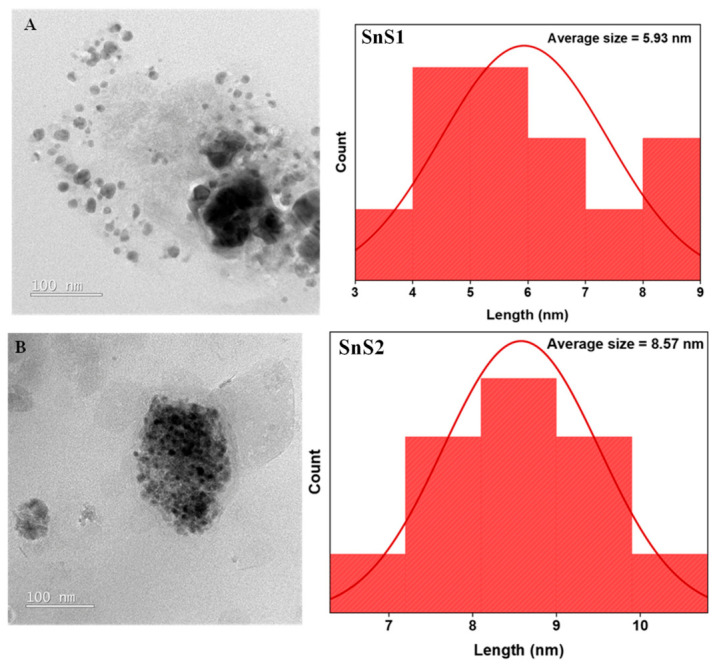

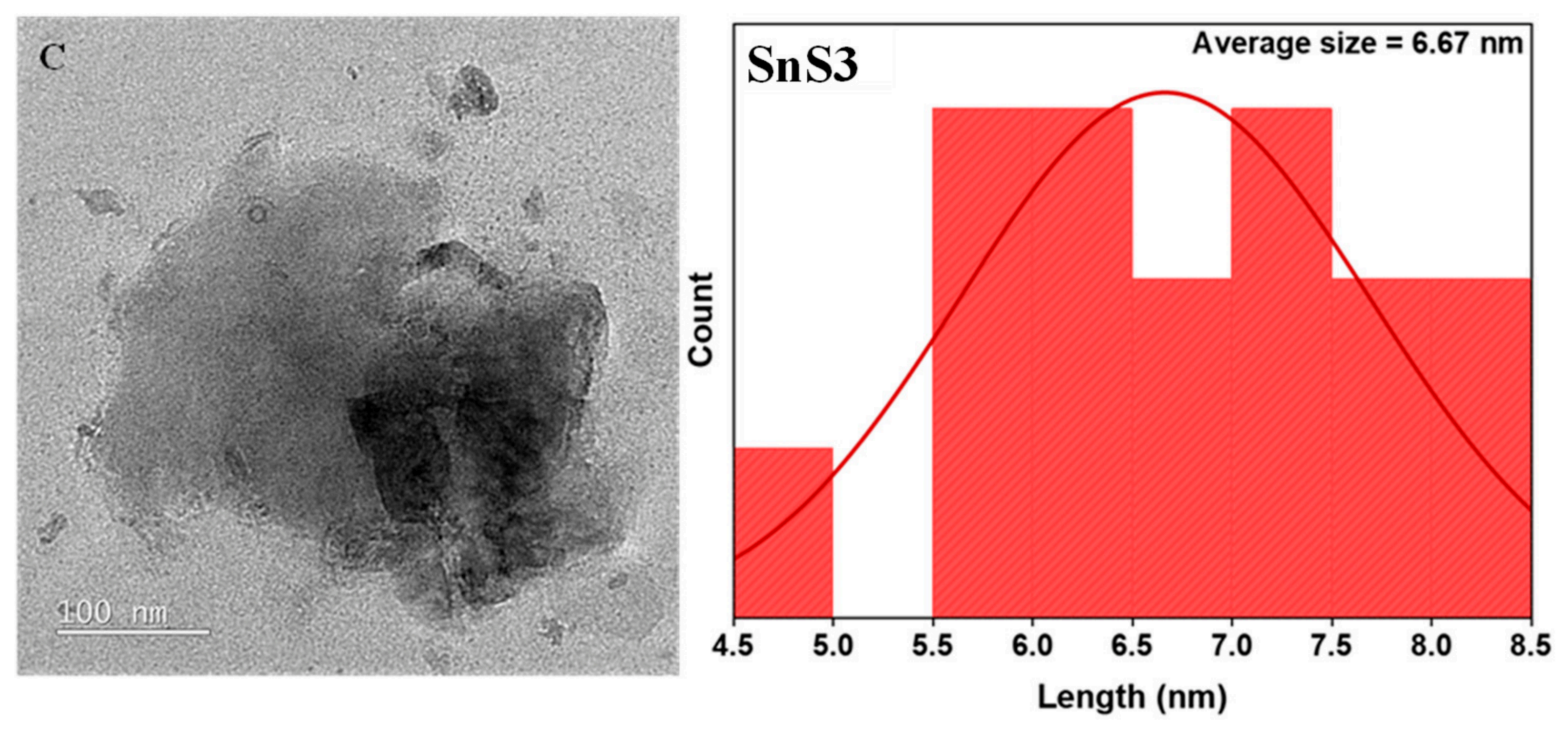
TEM micrographs and particle size-distribution plots of the SnS quantum dot: SnS1 (**A**), SnS2 (**B**), and SnS3 (**C**).

The TEM micrographs of SnS1, SnS2, and SnS3 (Figure 3A–C) reveal notable differences in particle dispersion and aggregation among the three samples. SnS1 displays relatively well-dispersed nanocrystals with a narrow size range, yielding an average particle size of 5.93 nm, as confirmed by the corresponding histogram (Figure 3A). In contrast, SnS2 exhibits a denser and more clustered morphology, consistent with its larger mean particle size of 8.57 nm, as shown in Figure 3B, suggesting enhanced nucleation and growth under its specific ligand environment [26,34]. SnS3 shows a broader distribution and partially fused particle domains, resulting in an intermediate average size of 6.67 nm, as shown in Figure 3C [26]. The size-distribution curves for all samples follow near-Gaussian trends, indicating reasonably uniform growth within each system. It should be noted that these values are approximate estimates, based on the observable nanoparticle features in the TEM images and should be interpreted cautiously, due to the limited resolution (100 nm scale bar) and possible particle agglomeration. The crystallite sizes estimated from XRD analysis were 28.02, 29.39, and 28.54 nm for SnS1, SnS2, and SnS3, respectively. The significantly larger crystallite sizes obtained from XRD compared to the TEM-derived particle sizes indicate that XRD probes larger coherent domains arising from particle aggregation and oriented attachment, while TEM measures individual nanoparticle dimensions. The crystallite sizes estimated from XRD and the particle sizes observed by TEM should not be expected to be identical, as the two techniques probe different physical characteristics of the material. XRD-based crystallite size (calculated using the Scherrer equation) reflects the average size of coherently diffracting domains and may be influenced by factors such as lattice strain, peak broadening, and instrumental effects [35,36]. In contrast, TEM provides a direct visualization of particle morphology, which can be affected by aggregation, limited contrast at low particle size, and image resolution [37,38]. Therefore, discrepancies between XRD-derived crystallite sizes and TEM-observed particle sizes are commonly reported in nanomaterials and should be interpreted with caution, as they reflect different physical aspects of the material. The TEM and XRD results collectively indicate that ligand chemistry strongly influences particle size, aggregation, and crystallinity, with SnS2 exhibiting larger and more compact nanocrystalline domains and SnS1 showing smaller, well-dispersed nanoparticles.

**Figure 4:**
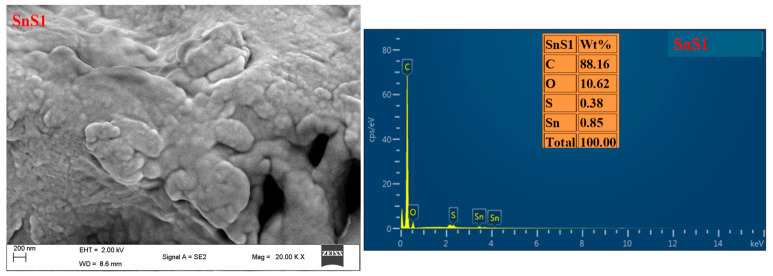

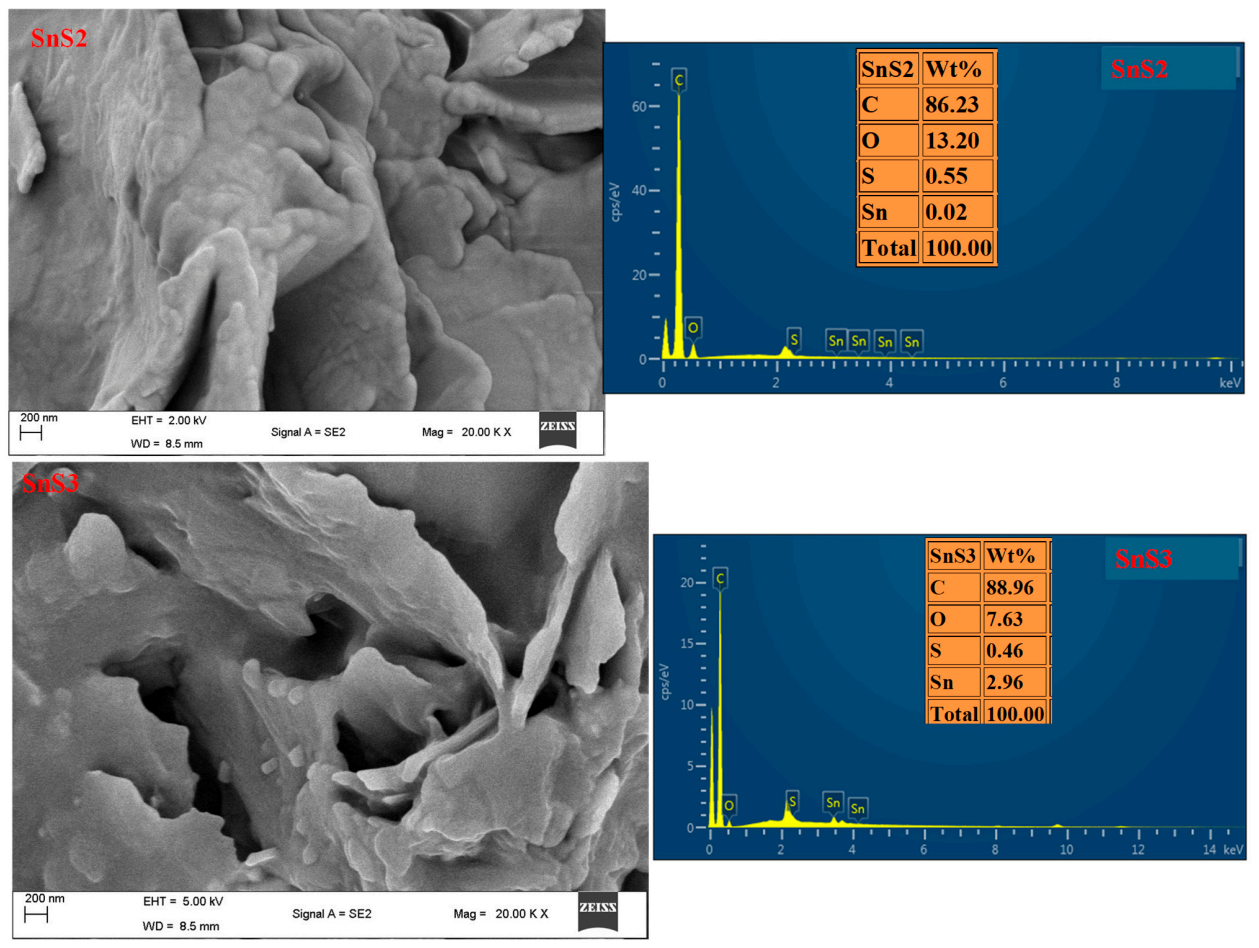
SEM images and EDS spectra of the SnS quantum dots: SnS1, SnS2, and SnS3.

The SEM micrographs, shown in Figure 1, reveal distinct morphological variations among the SnS samples. SnS1 appears as compact assemblies of relatively small platelets and nanoparticle clusters. In contrast, SnS2 exhibits more pronounced, larger layered and flake-like structures with sharper edges, indicating enhanced lateral growth. SnS3 displays a more open, wrinkled, and porous flake network. These morphological trends are consistent with the TEM and particle size distribution analyses, in which SnS2 shows the largest mean particle size, while SnS1 exhibits the smallest. Additionally, the corresponding EDS spectra confirms the presence of Sn and S in all samples, supporting the formation of SnS (Figure 4). However, the spectra are dominated by carbon and oxygen signals, which are attributed to residual organic ligands from the synthesis, surface-adsorbed species, and the carbon support used during SEM mounting [39,40]. Given the surface-sensitive nature of EDS and the use of organic capping agents, the elemental compositions are best interpreted qualitatively, rather than stoichiometrically [41,42]. Consequently, the combined SEM, TEM, and XRS analyses provide more reliable confirmation of the morphology and crystalline structure of the SnS nanostructures.

### 3.5. Optical Properties of SnS Quantum Dots

**Figure 5:**
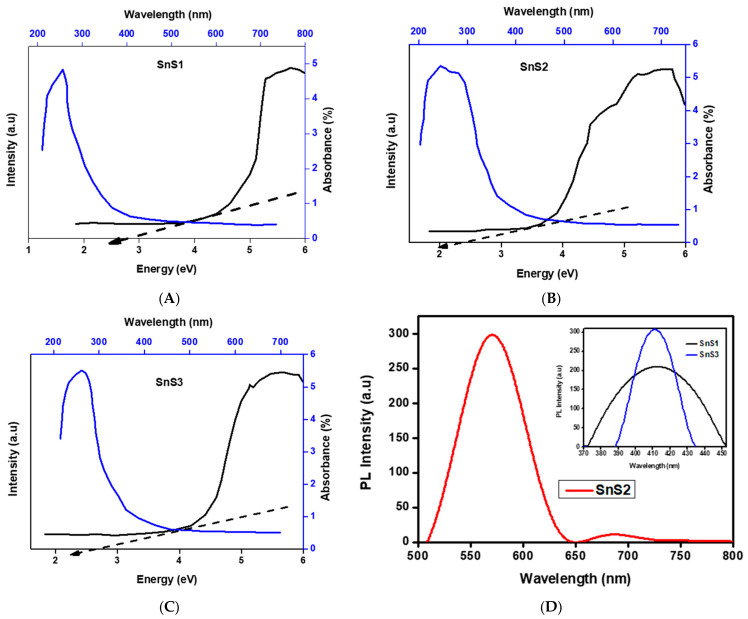
Absorption spectra and corresponding Tauc plots (**A**–**C**) and photoluminescence (PL) spectra (**D**) of SnS nanoparticles.

The absorption spectra and Tauc plots for SnS1, SnS2, and SnS3 are presented in Figure 5A–C. The optical band gaps were estimated using the Tauc relation, $\alpha h\nu =A(h\nu -E_{g}{)}^{n}$, where α is the absorption coefficient, hν is the photon energy, A is a constant, $E_{g}$ is the band gap energy, and n depends on the type of electronic transition [43]. SnS1 shows absorption in the 220–310 nm range with a band gap of 2.8, SnS2 absorbs between the 230–340 nm range with a reduced band gap of 2.2 eV, and SnS3 exhibits absorption from the 230–320 nm range with a band gap of 2.7 eV. The variation in band-gap values reflects differences in particle size, crystallinity, and surface effects, consistent with quantum confinement in SnS quantum dots [44,45]. The lower band gap and red-shifted absorption observed for SnS2 may be attributed to its relatively larger particle size, which weakens quantum confinement effects and enhances electronic delocalization. In addition to particle size, the nature of the capping ligands also contributes significantly to the optical properties of the SnS quantum dots. The ligands differ in their steric arrangement and electron-donating characteristics, due to the position of the methyl substituent on the aromatic ring [46]. These structural differences influence nanoparticle growth, surface passivation, and electronic interactions at the quantum dot surface. The methyl group present in m-toluidine and p-toluidine increases electron density on the ligand compared to aniline, which can modify ligand–surface interactions and affect charge-carrier distribution within the SnS quantum dots [47,48]. Furthermore, the photoluminescence (PL) spectra of SnS1, SnS2, and SnS3 (Figure 5D) exhibit distinct emission bands, further demonstrating size- and defect-dependent optical behaviour. SnS1 displays a broad emission band in the 370–450 nm region, whereas SnS2 shows a red-shifted emission extending from 510 to 640 nm, which may be associated with increased particle size and enhanced contributions from defect or surface-related states. In contrast, SnS3 exhibits emission in the 390–430 nm range, suggesting intermediate confinement effects. The broad nature of the PL peaks indicates the involvement of both band-edge transitions and defect-mediated recombination processes influenced by synthesis conditions and surface chemistry [49,50]. Overall, the combined absorption/Tauc and PL analyses confirm that the optical properties of SnS nanoparticles are strongly influenced by particle size, crystallinity, and surface states, demonstrating the tunability of absorption and emission behaviour through controlled synthesis.

### 3.6. Electrochemistry

**Figure 6:**
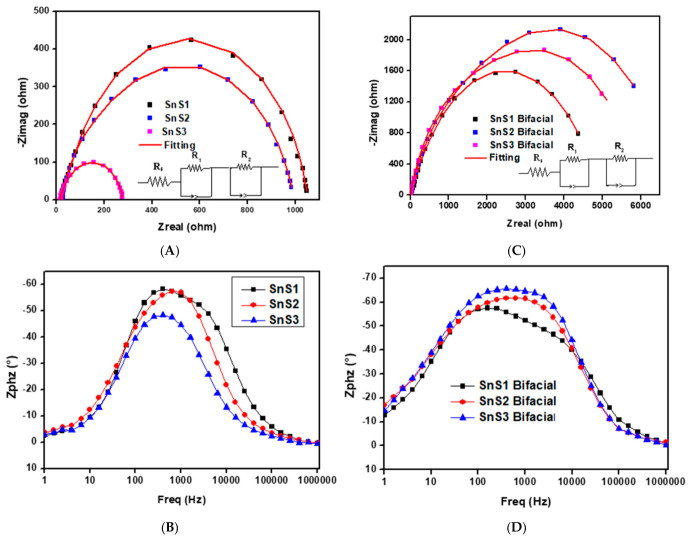
(**A**) Nyquist and (**B**) Bode plots of SnS nanoparticles; (**C**,**D**) show the corresponding bifacial electrode sides.

Electrochemical impedance spectroscopy (EIS) was employed to investigate the charge-transport, interfacial, and diffusion characteristics of SnS-based devices (SnS1, SnS2, and SnS3) under front-facing and bifacial configurations. The Nyquist and Bode-phase plots are shown in Figure 6, and the fitted equivalent circuit parameters are summarised in Table 2. The impedance spectra were satisfactorily fitted using an equivalent circuit. The impedance spectra were accurately modelled using an equivalent circuit consisting of a series resistance (Rs) and two parallel resistance–constant phase element branches (R‖CPE), representing Ohmic resistance, interfacial charge-transfer behaviour, and non-ideal capacitive processes associated with surface and structural heterogeneity [51,52].

The impedances of the resistor and the constant phase element are described by

(1) $$\begin{array}{cccc} & Z_{R}=R & & \end{array}$$

(2) $$\begin{array}{cccc} & Z_{\mathrm{CPE}}=\frac{1}{Q(j\omega {)}^{n}} & & \end{array}$$

where *Q* is the CPE magnitude and *n* (0 < *n* ≤ 1) represents the degree of deviation from ideal capacitive behaviour. The overall impedance of the equivalent circuit is expressed as

(3) $$\begin{array}{cccc} & Z\left(\omega \right)=R_{s}+\frac{R_{1}}{1+j\omega R_{1}CPE_{1}}+\frac{R_{2}}{1+j\omega R_{2}CPE_{2}} & & \end{array}$$

In these expressions, $R_{s}$ represents the series resistance arising from Ohmic losses within the FTO substrate, wiring, and electrolyte. The terms $R_{1}$ and $R_{2}$ correspond to the first and second charge-transfer resistances, respectively, each associated with distinct interfacial processes governing electron transfer and recombination at different regions of the device. The elements $CPE_{1}$ and $CPE_{2}$ denote the constant phase elements that account for non-ideal capacitive behaviour at these interfaces, typically caused by surface roughness, morphological heterogeneity, or distributions of reaction time constants. The term jω represents the complex frequency variable, where j is the imaginary unit and ω is the angular frequency applied during impedance measurements. Together, these elements describe the frequency-dependent impedance behaviour of the SnS devices, and enable separation of Ohmic, interfacial, and capacitive contributions to the overall electrochemical response.

In the front-facing configuration, all SnS devices exhibit well-defined semicircular behaviour in the Nyquist plots, indicating charge-transfer-controlled electrochemical processes [53]. SnS1 shows moderate series and interfacial resistances, suggesting balanced charge transport across the electrode–electrolyte interface. SnS2 exhibits increased interfacial resistance, reflected by a larger semicircle diameter and a shift in the Bode-phase peak towards lower frequencies, implying slower charge-transfer kinetics [54]. In contrast, SnS3 displays the smallest semicircle and the lowest overall impedance, indicating more favourable interfacial charge transport among the three devices.

Under bifacial operation, the impedance characteristics of the SnS devices change noticeably. The series resistance generally decreases, indicating improved charge collection from dual-side illumination. However, significant increases in the charge-transfer resistance are observed for SnS1 and SnS2, suggesting enhanced interfacial recombination or transport barriers, under bifacial conditions. SnS3 maintains comparatively lower interfacial resistance, reflecting more stable electrochemical behaviour when both sides of the electrode are active. Overall, the EIS analysis reveals that the electrochemical performance of SnS devices is governed by a combination of interfacial charge-transfer resistance and diffusion processes. SnS3 exhibits the most favourable charge-transfer properties, but is partially limited by diffusion resistance, whereas SnS2 is dominated by interfacial impedance. These findings highlight the strong influence of material morphology, ligand chemistry, and device configuration on the charge-transport dynamics of SnS-based energy-conversion devices. Together, the complementary EIS and PL analyses demonstrate that interfacial charge-transfer resistance and radiative recombination processes are strongly coupled in SnS devices, with material morphology and surface chemistry governing both optical emission and electrochemical response.

**Figure 7:**
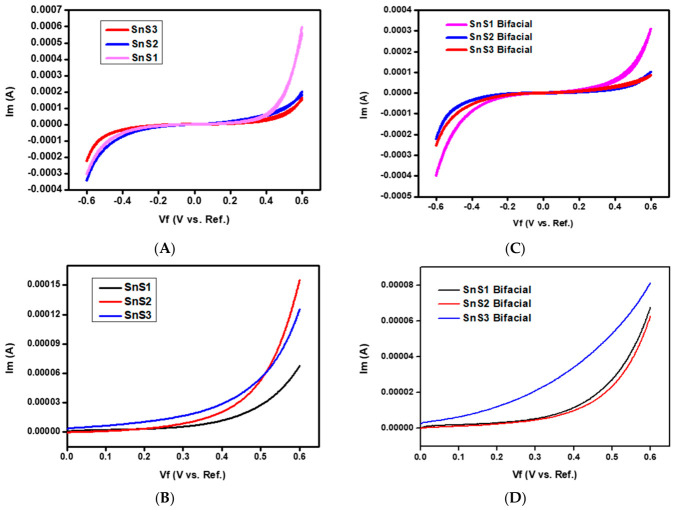
Cyclic voltammetry (**A**) and linear sweep voltammetry (**B**) of SnS nanoparticles; (**C**,**D**) represent the corresponding bifacial electrode sides.

The electrochemical behaviour of the SnS devices (SnS1–SnS3) was further investigated using linear sweep voltammetry (LSV) and cyclic voltammetry (CV), as shown in Figure 7. In the LSV curves, SnS3 exhibits the highest current response in the anodic potential region, followed by SnS2 and SnS1, indicating enhanced charge-transfer kinetics and a larger electrochemically active area for SnS3. This trend is consistent with the EIS results, where SnS3 showed comparatively lower charge-transfer resistance, facilitating more efficient carrier transport under applied bias. The CV profiles show quasi-rectangular shapes with weak redox features, suggesting predominantly capacitive behaviour dominated by surface-controlled electrochemical processes, rather than diffusion-limited faradaic reactions [55]. Among the three SnS, SnS3 displays the largest enclosed CV area, indicating superior charge-storage capability and faster interfacial charge dynamics, whereas SnS1 exhibits the lowest current density, in agreement with its comparatively higher interfacial resistance inferred from EIS analysis [51,54]. Under bifacial operation, an overall enhancement in current response is observed for all SnS devices, particularly in the anodic region. SnS3 bifacial exhibits the highest current density, confirming improved charge collection and transport when both sides of the electrode are active. The preservation of the relative performance order among the samples indicates that intrinsic material properties, rather than illumination geometry, primarily govern the electrochemical response. The LSV and CV results correlate well with both EIS and PL analyses. Samples exhibiting lower charge-transfer resistance and more efficient diffusion pathways display higher current responses, while stronger PL emission is associated with increased carrier recombination and comparatively reduced electrochemical currents [56]. This complementary relationship confirms that charge-transfer efficiency and recombination dynamics jointly dictate the electrochemical performance of the SnS devices.

**Figure 8:**
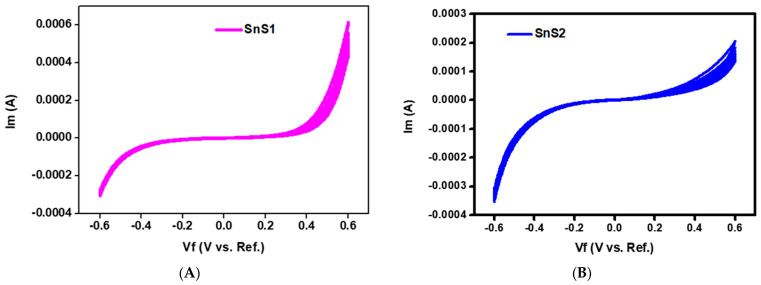

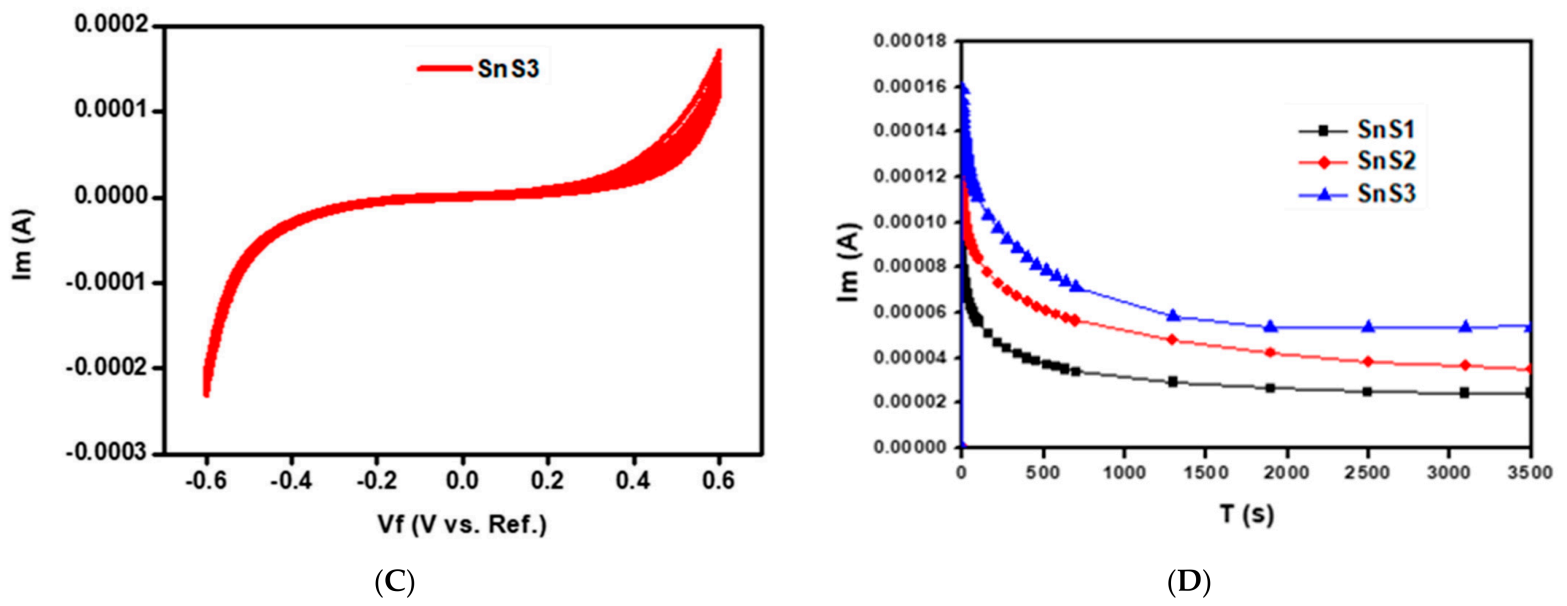
Multi-cycle cyclic voltammetry (CV (**A**–**C**)) and chronoamperometry (CA (**D**)) characteristics of monofacial SnS-based devices (SnS1–SnS3).

Multi-cycle cyclic voltammetry (CV) and chronoamperometry (CA) were employed to investigate the electrochemical charge-transfer behaviour, cycling stability, and time-dependent current response of the SnS-based devices (SnS1–SnS3) under applied bias, as shown in Figure 8. The multi-cycle CV curves of the SnS devices display reproducible current–potential profiles over successive scans, indicating stable electrochemical behaviour within the investigated potential window. The absence of pronounced peak shifting or severe current degradation suggests that the SnS electrodes maintain interfacial integrity during repeated cycling. Differences in current magnitude among SnS1, SnS2, and SnS3 reflect variations in interfacial charge-transfer kinetics, electronic conductivity, and surface-defect density. Devices exhibiting higher anodic and cathodic currents suggest more efficient charge transport across the SnS/electrolyte interface, whereas reduced current response indicates increased charge-transfer resistance and recombination losses. The smooth and quasi-rectangular shape of the CV profiles, together with limited hysteresis between forward and reverse scans, implies that the electrochemical response is dominated by capacitive and pseudocapacitive processes, rather than diffusion-limited faradaic reactions, which is consistent with nanostructured semiconductor electrodes [57,58]. Chronoamperometry measurements reveal a characteristic current–time behaviour for all SnS samples, consisting of a rapid initial decay followed by gradual stabilization at a steady-state current. The initial decay is attributed to capacitive discharge of the interfacial double layer, filling of surface and bulk trap states, and redistribution of charge carriers within the space-charge region [59]. This behaviour can be described by

(4) $$I\left(t\right)=I_{0}exp(-\frac{t}{\tau })+I_{ss},$$

where $I_{0}$ is the initial current, $I_{ss}$ the steady-state current, and τ the charge relaxation time constant. Among the SnS devices, variations in stabilization rate and steady-state current indicate differences in charge extraction efficiency and recombination dynamics. Devices sustaining higher steady-state current are indicative of improved interfacial charge-transfer properties and reduced recombination losses under prolonged bias conditions. The CA behaviour correlates well with the electrochemical impedance spectroscopy (EIS) results discussed earlier. SnS devices exhibiting lower charge-transfer resistance and reduced diffusion impedance show faster current stabilization and larger steady-state currents, whereas higher resistance values correspond to suppressed current response and slower stabilization. This correlation confirms that interfacial impedance and carrier-transport limitations play a dominant role in governing the electrochemical behaviour of SnS-based electrodes. In addition, the electrochemical response of the SnS devices can be related to their PL characteristics. Samples displaying stronger PL emission, typically associated with enhanced radiative recombination and defect-related transitions, tend to exhibit reduced electrochemical current and increased interfacial resistance [59]. Conversely, devices with reduced PL intensity demonstrate improved charge extraction and more stable electrochemical performance, indicating a favourable balance between radiative and non-radiative recombination processes. Overall, the combined multi-cycle CV and CA analyses demonstrate that the electrochemical performance of monofacial SnS-based devices is strongly influenced by interfacial charge-transfer resistance, carrier recombination dynamics, and surface states. The observed stability under repeated cycling and sustained bias highlights the suitability of SnS nanostructures for applications in photoelectrochemical and solar-energy conversion systems, where reliable charge transport and operational durability are critical.

### 3.7. J–V Curves (Front and Back Illumination)

**Figure 9:**
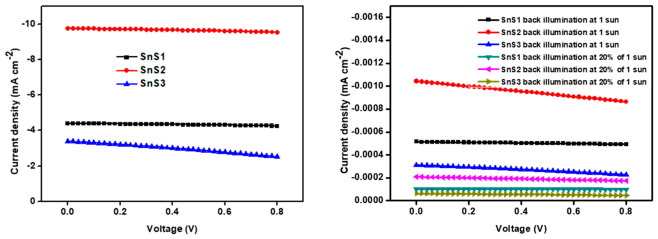
SCAPS-1D simulated J–V curves of SnS-based devices under monofacial and bifacial illumination conditions, using experimentally extracted SnS bandgaps.

The simulated current–voltage (J–V) characteristics of the SnS-based solar cell devices under front-side (1 sun) and rear-side illumination are presented in Figure 9. Under standard front illumination, the devices exhibit distinct J–V responses that reflect differences in the absorber bandgaps used in the SCAPS-1D simulations. SnS1 (2.7 eV) and SnS3 (2.8 eV) show higher open-circuit voltages due to their wider bandgaps, whereas SnS2, with a narrower bandgap of 2.2 eV, delivers a comparatively higher short-circuit current density as a result of enhanced photon absorption. This behaviour is consistent with fundamental photovoltaic principles, where wider bandgaps favour higher Voc but limit photocurrent generation, while narrower bandgaps enhance current at the expense of voltage. Under rear-side illumination corresponding to 20% of 1 sun, representing reflected solar radiation (albedo contribution), all devices exhibit an additional photocurrent contribution, leading to an upward shift of the J–V curves. Although the rear illumination intensity is significantly lower than that of front-side illumination, its contribution remains non-negligible, particularly for SnS2, whose smaller bandgap enables more effective utilisation of low-intensity transmitted and reflected photons. The reduced slope and lower current density observed under rear illumination are attributed to optical losses through the front layers and partial absorption in the electrode stack; however, the persistence of measurable photocurrent confirms the feasibility of bifacial operation. Overall, the J–V characteristics demonstrate that the absorber bandgap plays a decisive role in governing voltage and current trade-offs, while the inclusion of a 20% rear-illumination condition based on albedo realistically captures bifacial operating environments. These results validate the use of experimentally derived bandgaps in the numerical model and highlight the potential of SnS-based devices to benefit from bifacial configurations under real-world illumination conditions.

### 3.8. Photovoltaic Parameters

Table 3 presents a comparative analysis of SnS-based photovoltaic devices fabricated experimentally in this work, alongside selected literature reports. The SCAPS-1D simulations were parameterized exclusively using the experimentally measured bandgaps of the SnS absorbers (2.7, 2.2, and 2.8 eV for SnS1, SnS2, and SnS3, respectively), ensuring that the numerical predictions reflect realistic material properties. This approach allows direct assessment of how computationally optimized architectures translate into experimentally achievable device performance. Experimentally, the monofacial devices exhibit PCEs of 3.40%, 2.03%, and 7.63% for SnS1, SnS2, and SnS3, respectively. The performance limitations are primarily associated with trap-assisted recombination, series resistance, and non-ideal interfacial contacts. In addition, the broadened emission bands observed in the PL spectra suggest the presence of surface defects and localized trap states within the SnS thin films. These defect states can act as non-radiative recombination centres, leading to increased carrier losses and reduced charge-extraction efficiency, which may contribute to the lower experimental efficiencies compared to the SCAPS-1D simulated results. Among the fabricated devices, SnS3 delivers the highest efficiency, consistent with its favourable bandgap of 2.8 eV, which supports improved charge transport and optimal photovoltage generation. The comparatively improved performance of SnS3 also suggests reduced recombination losses and improved interfacial charge transfer relative to SnS1 and SnS2. The observed variations in Voc and Jsc across the devices correlate with their respective bandgaps, highlighting the intrinsic trade-off between photocurrent and photovoltage in SnS absorbers. Meanwhile, devices in the literature employing alternative transport layers, such as PbS/Ni- and WS_2_-based architectures, report relatively high efficiencies, exceeding 22%, supporting the validity of simulation-driven optimisation strategies for SnS absorbers.

Under bifacial illumination, the devices show PCEs of 3.40%, 2.03%, and 7.63% for SnS1, SnS2, and SnS3, respectively. The increased photocurrent under bifacial operation is limited by optical and interfacial losses, indicating that practical bifacial gains remain constrained by material and interface quality. Overall, the results in Table 3 demonstrate that experimentally informed SCAPS-1D modelling using actual bandgap values can identify high-efficiency SnS device architectures, while experimental performance is constrained by fabrication and interface limitations. The consistent trends between simulation and experiment, and the literature, highlight the potential of SnS absorbers for monofacial and bifacial photovoltaic applications, provided recombination and resistive losses are further minimized.

## 4. Conclusions

The experimental evaluation of SnS quantum dot-sensitized solar cells confirms that ligand-controlled growth strongly influences crystallographic quality, optical bandgap, and photovoltaic performance under both monofacial and bifacial operation. Although all samples were crystallized in the orthorhombic SnS phase, their functional behaviour differed markedly, as reflected in the variation in particle size (5.93–8.57 nm) and bandgap (2.2–2.8 eV). These differences translated directly into device performance, where SnS3, with an intermediate bandgap of (2.7 eV) and particle size of (~6.67 nm), delivered the highest efficiency of 7.63%, significantly outperforming SnS1 (3.40%) and SnS2 (2.03%). In contrast, the larger particle size and lower bandgap of SnS2 did not result in improved efficiency, indicating that enhanced light absorption alone is insufficient without effective charge transport. Furthermore, the limited improvement under bifacial illumination suggests that recombination and interfacial losses restrict the effective utilization of additional photogenerated carriers. Overall, the results demonstrate that optimal device performance is achieved through a balance of structural and optoelectronic properties, highlighting ligand-controlled precursor design as a key factor in tuning SnS quantum dots for photovoltaic applications.

## Figures and Tables

**Table 1:** XRD peak positions, d-spacings, and crystallite sizes of SnS1–SnS3.

Peak No.	Hkl	2θ (°)	d [Å]	FWHM (°)	D (nm)
SnS1	(120)	25.19	3.5413	0.2708	30.08
	(021)	26.38	3.3760	0.3380	24.16
	(111)	31.09	2.8747	0.2738	30.14
	(040)	34.27	2.6148	0.4388	18.96
	(131)	37.61	2.3899	0.2695	31.17
	(151)	51.51	1.7773	0.2627	33.60
					Average (nm) 28.02
SnS2	(120)	24.92	3.5784	0.5765	14.12
	(021)	26.59	3.3578	0.2626	31.11
	(111)	30.35	2.9503	0.2205	37.36
	(040)	34.44	2.6084	0.4202	19.81
	(131)	37.32	2.4134	0.2620	32.03
	(151)	51.13	1.7894	0.2104	41.89
					Average (nm) 29.39
SnS3	(120)	25.12	3.5420	0.2762	29.79
	(021)	26.47	3.3727	0.3694	22.11
	(111)	30.93	2.8892	0.2786	29.61
	(040)	33.67	2.6661	0.2961	28.05
	(131)	37.61	2.3894	0.2768	30.34
	(151)	51.50	1.7773	0.2789	31.65
					Average (nm) 28.54

**Table 2:** Fitted EIS parameters (*Rs*, *R*_1_, and *R*_2_,) for monofacial and bifacial SnS devices.

Sample	*Rs* (Ω)	*R*_1_ (Ω)	*R*_2_ (Ω)
SnS1	28.57	20.98	1006.36
SnS2	26.16	79.96	887.52
SnS3	16.13	6.05	257.06
SnS1 (Bifacial)	19.35	2105.31	49.10
SnS2 (Bifacial)	19.28	60.68	1782.15
SnS3 (Bifacial)	11.01	8.16	440.39

**Table 3:** Comparison of simulated and experimental photovoltaic parameters of SnS-based monofacial and bifacial solar cell devices investigated in this work, alongside representative SnS-based devices reported in the literature.

Device	Type		V_oc_ (V)	J_sc_ (mA cm^−2^)	*FF* (%)	PCE (%)	Ref.
FTO/SnS_2_/PbS/Ni	Monofacial	Simulation	0.99	26.99	85.08	22.96	[60]
ZnO/ZrS_2_/SnS/Au			0.86	34.2	70.0	21.0	[61]
FTO/WS_2_/SnS_2_QDs/SnS/Pt			0.95	26.38	84.95	22.3	[62]
ITO/ZnO/ZnS/CFTS/SnS/Au			0.70	32.53	61.98	14.20	[63]
ZnO/ZrS_2_/SnS			0.70	42.32		21.78	[61]
FTO/SnS_2_/SnS1/CsSnI_3_/Se		Experimental	-	4.39	-	3.40	This work
FTO/SnS_2_/SnS2/CsSnI_3_/Se			2.852	3.39	20.95	2.03	This work
FTO/SnS_2_/SnS3/CsSnI_3_/Se			-	9.76	-	7.63	This work
SLG/Mo/SnS/ZnxCd1-xS/i-ZnO/AZO/Ag			0.291	-	-	2.98	[60]
SLG/Mo/SnS/CdS/i-ZnO/AZO/Ni/Ag			0.405	22.86	0.401	3.72	[64]
FTO/SnS/CdS/ZnO/ITO	Bifacial	Experimental	0.30	9.8	40.0	1.2	[65]
Al/FTO/SnS_2_/SnS1/CsSnI_3_/Se			-	42.59	-	3.40	This work
Al/FTO/SnS_2_/SnS2/CsSnI_3_/Se			3.684	85.82	38.99	2.03	This work
Transparent TPV (FTO/Ga_2_O_3_/SnS/AgNW)			0.526	2.40	56.5	7.13	[66]
Al/FTO/SnS_2_/SnS3/CsSnI_3_/Se			1.294	32.9	22.78	7.63	This work

## Data Availability

The data used in this work is available upon reasonable request to the authors.
